# Fucoidans in Nanomedicine

**DOI:** 10.3390/md14080145

**Published:** 2016-07-29

**Authors:** Lucas Chollet, Pierre Saboural, Cédric Chauvierre, Jean-Noël Villemin, Didier Letourneur, Frédéric Chaubet

**Affiliations:** 1 Inserm, U1148, LVTS, University Paris Diderot, X Bichat Hospital, F-75877 Paris, France; lucas.chollet@algues-et-mer.com (L.C.); pierre.saboural@univ-paris13.fr (P.S.); cedric.chauvierre@inserm.fr (C.C.); didier.letourneur@inserm.fr (D.L.); 2 Galilée Institute, University Paris 13, Sorbonne Paris Cité, F-93430 Villetaneuse, France; 3 Algues & Mer, Kernigou, F-29242 Ouessant, France; jn.villemin@algues-et-mer.com

**Keywords:** fucoidans, nanomedicine, sulfated polysaccharides, nanosystems, drug delivery, imaging agent, tissue regeneration

## Abstract

Fucoidans are widespread cost-effective sulfated marine polysaccharides which have raised interest in the scientific community over last decades for their wide spectrum of bioactivities. Unsurprisingly, nanomedicine has grasped these compounds to develop innovative therapeutic and diagnostic nanosystems. The applications of fucoidans in nanomedicine as imaging agents, drug carriers or for their intrinsic properties are reviewed here after a short presentation of the main structural data and biological properties of fucoidans. The origin and the physicochemical specifications of fucoidans are summarized in order to discuss the strategy of fucoidan-containing nanosystems in Human health. Currently, there is a need for reproducible, well characterized fucoidan fractions to ensure significant progress.

## 1. Introduction

**Figure 1:**
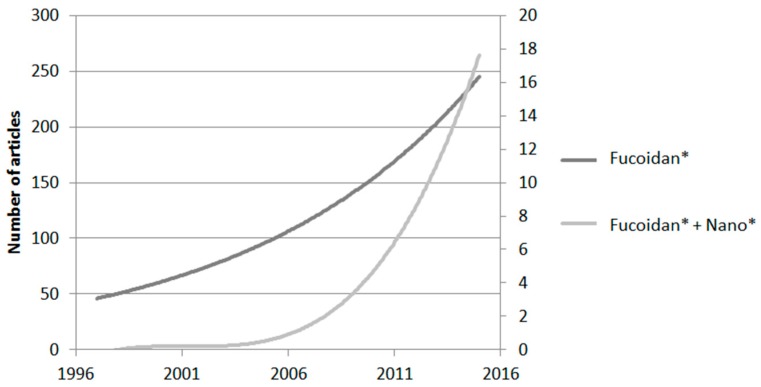
Evolution of published articles reporting fucoidans (from Web of Science). Left axis: number of articles for “Fucoidan*”, right axis: number of articles for “Fucoidan* + Nano*”.

Fucoidans are abundant cost-effective marine polysaccharides which exhibit a wide spectrum of biological activities with potential clinical applications. For more than half a century, extensive works have been published about the activities of these molecules; some of the most recent reviews are listed in Table 1. Recently, nanomedicine began to incorporate the use of fucoidans especially in the domains of cancer, regenerative medicine, and cardiovascular diseases, fields in which nanotechnologies are making progress every day. Since 2005, reports on fucoidans in nanomedicine have increased to represent about 7% of the overall works in 2014 related to both topics (Figure 1).

This review focuses on the progress at the interface of fucoidans and nanomedicine in the perspective of development of new diagnostic and therapeutic tools for human use. In the first part, fucoidans and their biological properties are briefly presented and in the second part the main studies of fucoidans with regard to developments in nanomedicine are given. In the last part, we discuss the relevance of these studies in light of the structural data of fucoidans and we question an appropriate strategy for the development of fucoidans for human applications.

## 2. What Are Fucoidans?

Fucoidans belong to a large family of marine sulfated polysaccharides named fucans mainly constituted of sulfated l-fucose, which include also ascophyllans (xylofucoglycuronan and xylofucomanuronan) and sargassans (glycuronofucogalactan) [1,2]. Fucoidans were first discovered in 1913 by Kylin in brown algae: *Ascophyllum nodosum*, *Fucus vesiculosus*, *Laminaria digitata* and *Laminaria saccharina* [3]. Since then, fucoidans have been identified in 70 more species of brown algae (*Phaeophyceae*) [4,5,6,7,8,9,10,11,12], in the body wall of some marine invertebrates such as sea cucumber (*Holothuroidae*), and in the egg jelly coat of sea urchins (*Echinoidea*) [4,13,14].

Fucoidans are contained in the extracellular matrix (ECM) of brown algae’s cell walls [1]. Considering the eco-physiological influences (alga species, location and season of harvesting, the position on the intertidal zone, etc.) on the composition of fucoidans, they are implicated in the ionic and osmotic regulation and in the mechanical support of the cell wall [2,15]. Thus, the algae which have been most exposed to drying seem to contain the highest fucoidan content. In sea urchin, fucoidans play a role in the fertilization process since they are found in the surrounding coating of the female gamete (zona pellucida) and participate in the species-specific acrosome reaction [16,17]. In sea cucumber, fucoidans could be involved in the structural support of the body wall in the saline environment, as for algae [18].

**Figure 2:**
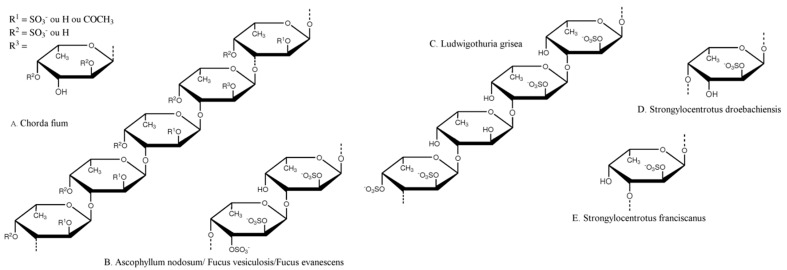
Repeating chemical structures of some fucoidans from brown algae (**A**) *Chorda filum* [28]; (**B**) *Ascophyllum nodosum*, *Fucus vesiculosus,* and *Fucus evanescens* [22,23,31] and from marine invertebrates: sea cucumber (*Holothuriodea*) (**C**) *Ludwigothuria grisea* [29]; (**D**) *Strongylocentrotus droebachiensis* [17], and (**E**) *Strongylocentrotus franciscanus* [30].

The chemical composition of fucoidans is extremely variable depending on eco-physiological parameters. The first structure was elucidated in 1950 by Conchie and Percival from a fucoidan extracted from *Fucus vesiculosus* [19]. Kloareg et al. determined that fucoidans were composed of 50%–90% of l-fucose, 35%–45% of sulfate and less than 8% of uronic acid with a linear backbone based on an α(1→2)-glycosidic linkage of O-4 sulfated l-fucose and some oses like galactose, mannose, xylose, and glucose [1,2]. In 1993, Patankar et al. published a revised structure of a commercial fucoidan from *F. vesiculosus*: mainly an α(1→3)-l-fucose linear backbone with sulfate substitution at O-4 and some α-l-fucose branched at O-4 or O-2 [20]. Thereafter, studies on fucoidans’ structure evidenced different repeating units for highly purified fucoidan fractions from different species [21,22,23,24,25,26,27,28]. Structures are based on an α(1→3)-l-fucose backbone with some alternating α(1→4) linkages. The sulfation patterns are variable but sulfate groups are mainly found at O-2 and O-4 [29,30]. Fucoidans extracted from marine animals have a more regular chemical structure (Figure 2).

There are almost as many methods of extraction of fucoidans from brown algae as there are studies dealing with these polysaccharides. However a general pattern can be proposed: a first extraction with organic solvents (e.g., acetone, toluene, etc.) from the fresh materials provides dried extracts which can be treated with methanol, ethanol or formaldehyde to remove hydrophobic compounds like dyes and lipids. The remaining alginates are precipitated with calcium, followed by acidic and sometimes alkaline hydrolyses at temperatures ranging from ambient up to 100 °C, enabling both to discard non-fucoidan polysaccharides (in particular laminarin) and decrease the molecular weight of the fractions. More recently, microwave assisted extractions have been developed [32]. The extraction conditions influence the final chemical composition of the fucoidan fractions [9,12] which remain complex mixtures of macromolecular species with large molecular weight distributions (100–1000 kDa). Although it is now widely admitted that the term “fucoidan” refers to a sulfated-l-fucose based polymer, it is still not possible to speak of a single compound; “fucoidans” should always be used as a generic term as was first proposed by Larsen in 1966 [33] and a fraction specifically prepared should be referred to as “a fucoidan fraction”(FF). Both terms will be used in this review.

The bioactivities of low molecular weight FF were found to mimic those of heparin, a glycosaminoglycan of animal origin with a molecular weight of about 15 kDa. As a consequence, depolymerization methods of raw fucoidans were developed: by acid hydrolysis [9], by radical cleavage [34], by enzymatic degradation (fucoidanases) from bacteria as well as digestive secretion of mollusk [35,36,37,38,39], and by gamma irradiation [40,41,42]. These methods could often cause structural alteration (like debranching and desulfation) likely affecting the biological activities. An alternative approach to extraction methods is the synthesis of FF, either with enzymes or through a full chemical process. Fucoidanases, enzymes extracted from marine invertebrates, marine fungi or bacteria, are able to selectively degrade the fucose-based backbone of fucoidans offering structurally well-defined and biologically active fragments. Silchenko et al. isolated several fucoidanases [37,43] and developed a method for the screening and the detection of these enzymes in bacterial colonies [39]. Nifantiev et al. achieved the chemical synthesis of oligofucosides up to hexadecafucosides [44,45] with controlled sulfation patterns, allowing different types of FF to be obtained: some fractions were built up of (1→3)-linked α-l-fucose residues similar to the one found in *Laminaria saccharina* [24,27] or *Chorda filum* [28] and others were built up of alternating (1→3)- and (1→4)-linked α-l-fucose residues as found in *Ascophyllum nodosum* or *Fucus evanescens* as examples. These bottom-up approaches could be used to synthesize a wide range of FF with well-defined structures, improving the knowledge in the structure-biological activity relationships for these molecules. Although, tremendous progress in glycobiology and glycomedicine has driven the development in oligosaccharide synthesis [46], either with the aid of enzymes or by full synthesis, industrial preparation of tailor-made FF remains still hard to achieve due to low overall yields and the time needed to complete the process. Interestingly, there is currently no standard method to obtain reproducible bioactive well defined FF either from top-down or bottom-up strategies.

## 3. Biological Properties of Fucoidans

The interest of the scientific community in fucoidans and their low molecular weight fractions (i.e., below 30 kDa) is mainly driven by the wide spectrum of biological activities evidenced from their discovery up to now. Table 1 gathers the main biological effects reported and the identified targets. Over the last decades, new functions of polysaccharides and more specifically low molecular weight (LMW) fractions have attracted the interest of scientists for their ability to act in a wide variety of biological processes [47]. Structural variations such as degrees of substitution with chemical groups (in particular carboxylates, acetates or sulfates) are implicated in biological responses [48,49] and their activities are often attributed to their negative charges and sulfation degrees rather than to any specific carbohydrate structure as described for heparin [50]. Low molecular weight fractions from mammalian, glycosaminoglycans (GAGs) and more particularly low molecular weight-GAGs from heparin, heparan sulfate, hyaluronate, and chondroitin sulfate are implicated in a wide variety of biological processes as cofactors for growth factor, cytokines and chemokines production, tumorigenesis, signaling molecules in response to infection or other cellular damage, regulator of blood coagulation, and assisting viral and bacterial infections [51,52,53], the most active compounds being neutral or anionic structures partially acetylated or sulfated.

So far, multiple targets have been identified in blood and tissues to explain the biological activities of fucoidans. The anticoagulant activity, one of the most studied with reference to heparin, can be explained by the interactions of fucoidans towards natural thrombin inhibitors, serpins antithrombin, and heparin cofactor II, enhancing their activity [11]. P- and L-selectins, membrane proteins which play a role in the leukocyte rolling and extravasation process in vascular inflammatory response, have been reported and studied as the main targets in the anti-inflammatory activity of fucoidans [54,55]. Likewise, the inhibition of complement activation through classical and alternative pathways, also responsible for fucoidans anti-inflammatory activity, occurs by inhibiting formation or function of several complement’s enzymes such as C4, C4b,2a, C3, and C3b,Bb [56].

Antiviral activity is ensured by the binding of fucoidans to the CD4 glycoprotein on T lymphocytes, an essential immunoglobulin in the infection process of host cells by the viruses [67]. Fucoidans, especially fucoidans with high sulfation content, inhibit α-glucosidase and α-amylase, two digestive enzymes, increasing or interrupting the absorption delay of glucose. The most sulfated fractions have an inhibitory effect more pronounced than the less sulfated ones and electrostatic interactions are likely involved [112,113]. In tissues, fucoidans have an effect on several enzymes responsible for mitosis or cellular apoptosis such as caspases-3, -8 and -9 or mitogen-activated protein kinase (MAPK) and their inhibitors [91,92,102], enhancing or silencing these factors in opposite ways in cancer cells or healthy cells (protective effect). Furthermore, LMW fucoidan fractions inhibit the accumulation of hypoxia-inducible factors-1 (HIF-1) which promote tumor angiogenesis in cancer cells [99].

The biological activities of fucoidans seem mainly modulated by their molecular weight and their sulfate content, which, as previously stated depend on the starting material and the method of preparation. One of the most striking examples is the anti/pro-angiogenic activity. Pomin et al. evidenced that fucoidans of various origins exhibit an anti-angiogenic activity due to their ability to interfere with vascular endothelial growth factors (VEGFs) and basic fibroblast growth factor (FGF-2) [11]. However, Matou et al. showed the pro-angiogenic effect of fucoidans, also extracted from *Ascophyllum nodosum*, by enhancing the expression of α6, β1, and PECAM-1 integrin subunits on the surface of endothelial cells, resulting in an increase of FGF-2-induced angiogenesis [85]. Nifantiev et al. reviewed numerous studies on the angiogenic activities of fucoidans from different brown algae to highlight structure-activity relationships. They could only conclude that FF from *Ascophyllum nodosum* with MW over 30 kDa exhibited anti-angiogenic activity whereas FF with MW lower than 30 kDa exhibited pro-angiogenic activity [10].

Fucoidans exhibit several bioactivities against a wide spectrum of pathological situations with a remarkable absence of adverse effects. On one hand, it is now widely accepted that levels of l-fucose and sulfate as well as the molecular weight are major structural parameters whose variation affect the biological properties. On the other hand, each algae species produces its own type of fucoidan whose composition also depends on the conditions of obtaining. Pharmaceutical grade fucoidans with well-defined molecular weight distributions and thoroughly defined chemical compositions are now needed. It is necessary to obtain proper structure-activity relationships in order to select the most relevant FF for human clinical trials.

## 4. Fucoidans in Nanomedicine

Nanomedecine, also defined as nanotechnology in the biomedical field, has gained considerably in interest in the last decade. Nanosystems, such as, in a non-exhaustive way, nanoparticles, polymeric carriers, nanotubes, micelles, and liposomes have size-dependent properties and nanometer-scale dimensions which play important roles in biological systems. For half a century, they have been developed for therapeutic and diagnostic purposes and more recently have found tremendous applications in regenerative medicine with the development of nanostructured biocompatible scaffolds for cell organization and proliferation [142]. Moreover, nanotheranostics or theranostic nanomedicines have also been developed combining diagnosis and therapy to monitor both the release and the bioavailability of the drug at the proper pathological site [143]. The major interest of nanomedicine remains for drug delivery and personalized medicine defined as “the right drug to the right patient at the right moment” [144,145]. Most of these new biomedical tools are currently employed for treatments via oral or parenteral administration to fight cancer, iron deficiency or multiple sclerosis as examples [142]. Lovrić et al. reviewed the marketed products and those with the greatest potential [142].

Sulfated polysaccharides, especially fucoidans have been included in nanosystems for diagnostic, drug delivery, and tissue engineering [146,147]. Fucoidans have also been used as stabilizers of nanoparticles (NPs) [148,149,150,151,152] or to study the behavior of the aqueous suspension of chitosan/fucoidan-based NPs [153,154,155]. These works will not be detailed here since this review is dedicated to FF-containing nanosystems with direct applications to diagnosis and therapy. Table 2 assembles such applications, mainly with fucoidan-containing nanoparticles (FNPs), and the most relevant are explained in the following text. Table 3 indicates the origin and physicochemical data of FF used in these 31 reported studies.

### 4.1. Fucoidans in Therapeutic Nanosystems

In 2006, Sezer and Akbuga were the first to design FNPs named “fucospheres” from mixtures of fucoidan and chitosan for drug delivery purposes [163]. Two years later, they demonstrated the efficacy of fucospheres from the same origin over chitosan-based NPs in the treatment of dermal burns in rabbits [189,190]. The fucospheres size ranged from 300 nm to 1000 nm with surface charges from +6 to +26 mV and were tested in vitro on freshly excised chicken back skin. Then, in vivo tests were conducted on rabbits with the most efficient FNPs and the authors observed the highest level of wound healing after 21 days in groups treated with fucospheres as compared to those treated with chitosan microspheres or FF solution. FF has been found to accelerate the healing effects on dermal burns when coupled with chitosan which is able to re-epithelize and encourage fibroblast migration to the burn sites.

At the same time, Nakamura et al. designed FF/chitosan microparticles loaded with fibroblast growth factor 2 (FGF-2) [164]. FF was purified from the starting material with calcium chloride. FGF-2-loaded microparticles were then subcutaneously injected and neovascularization was observed in ischemic tissue in a mice model.

In 2013, another group synthesized FGF-2-loaded spherical nanoparticles, by dripping a mixture of FF and FGF-2 into a solution of chitosan under stirring [165]. This study evaluated the release of the growth factor in vitro and its effect on the differentiation of PC12 neural progenitor cells evidencing a synergistic activity on nerve cell growth as compared to FGF-2 in solution alone.

Chitosan/FF/tripolyphosphate NPs were synthesized and loaded with stromal cell-derived factor-1 (SDF-1) as a therapeutic agent for tissue regeneration by Huang et al. [166]. FNPs were efficient in protecting SDF-1 from inactivation by proteolysis, heat, and pH and the released SDF-1 was able to improve the proliferation and the migration of rat mesenchymal stem cells for up to seven days.

In 2009, Sezer et al. also used fucospheres to encapsulate and to deliver plasmid DNA encoding GM-GSF [179]. The diameter ranged from 150 to 400 nm with a zeta potential from 8.3 mV to 17.1 mV depending on the chitosan molecular weight. The encapsulation capacity was evaluated between 84% and 95% depending on the chitosan molecular weight and the amount of plasmid added to the loading solution. Once encapsulated in fucospheres, the plasmid was released in vitro and its integrity was validated. No tests on cells or in vivo experiments have been published yet.

The same year, Kurosaki et al. developed a ternary complex FF/pDNA/Polyethylenimine [180]. The complexes had 72 nm mean diameter and −27 mV zeta potential. FNPs were tested on B16-F10 mouse melanoma cells to assess the uptake and the transfection efficiency in vitro. They showed no cytotoxicity as compared to the pDNA/PEI NPs after 2 h of incubation and a concentration of 10 mg/mL of pDNA. However, when added to the B16-F10 cells, FNPs showed significantly lower uptakes and gene expression as compared to fucoidan-free NPs.

Pinheiro et al. synthesized chitosan/fucoidan multilayer nanocapsules (FNCs) as a vector for the controlled release of poly-l-lysine (PLL), a polypeptide exhibiting strong antimicrobial activity, as a drug model [176]. Ten chitosan/fucoidan layers were formed over a polystyrene core removed after synthesis by repeated dipping in THF. The encapsulation of PLL was better when performed during the formation of the NCs. The encapsulation efficiency and the loading capacity of FNCs strongly depended on the initial PLL concentration used, with the highest values obtained at a PLL concentration of 1 mg·mL^−1^. PLL release from the FNCs was found to be pH-dependent with a maximum at pH 2 due to a weakening of the nanocapsules interpolyelectrolyte structure and suggested a peculiar release behavior. Due to the bioactivities and non-cytotoxicity of FF and chitosan, FNCs were envisaged by the authors as nanocarriers to protect and release bioactive compounds for food and pharmaceutical applications.

Yu et al. prepared chitosan-based beads embedded with FNPs for oral delivery of berberine, an antimicrobial agent used to inhibit the growth of bacteria in the digestive system [168]. The NPs/beads complexes inhibited the growth of *Staphylococcus aureus* and *Escherichia coli* in simulated gastric or intestinal fluids. Complexes also demonstrated a delayed drug release over 24 h in simulated gastric fluid, which could be suitable for later drug delivery to the small intestine. Another group developed chitosan/fucoidan-taurine conjugate NPs to deliver berberine via the oral route to treat defective intestinal epithelial tight junction barrier [175]. The release of berberine was found to be pH-dependent with higher release at intestinal pH (7.4) than gastric pH (2.0). In vitro, the authors demonstrated the protective effect of the FNPs on Caco-2 cell monolayer, as a model of the epithelial barrier, co-cultured with LPS-treated RAW 264.7 cells. The results suggested the utility of such FNPs in allowing local delivery of berberine on bacterial-derived lipopolysaccharides intestinal epithelia tight junction disruption, to restore barrier function in inflammatory and injured intestinal epithelium.

Huang et al. developed antioxidant FNPs for antibiotic delivery to the lungs [169]. The use of FF was explained by their antioxidant and anti-inflammatory properties in order to treat pulmonary allergic inflammations. FNPs size ranged from 230 nm to 250 nm and their compactness and stability were maintained for 25 days. They exhibited highly potent antioxidant effects by scavenging 1,1-diphenyl-2-picrylhydrazyl (DPPH), and reducing the concentration of intracellular reactive oxygen species (ROS) as well as superoxide anion in stimulated macrophages. As an antibiotic model drug, Gentamicin (GM) was used for controlled release assays in vitro. The FNPs released 99% of GM over 72 h after an initial 10 h burst release. They were considered as potential carriers for antibiotics delivery to the lungs in the case of pulmonary infections and to be useful to treat airway inflammatory diseases.

In order to deliver drugs with low solubility and high pH sensitivity, Huang et al. developed *O*-carboxymethyl chitosan/fucoidan NPs to increase cellular curcumin uptake (Cur), a polyphenolic compound exhibiting several biological activities such as antitumor, antioxidant, inhibiting cardiovascular diseases, and inducing apoptosis [174]. Cur-loaded FNPs (Cur-FNPs) had an average diameter of 270 nm and encapsulated 92.8% of the drug. Cur-FNPs considerably decreased the cytotoxicity of Cur to mouse fibroblasts cells (L929), were stable in the gastric environment (pH 2.5), and allowed the release of Cur in the simulated intestinal environment (pH 7.4). The cellular uptake of Cur-FNPs was evaluated using Caco-2 cells. An internalization of Cur-FNPs by the cells through energy-dependent endocytic pathways was observed making *O*-carboxymethyl chitosan/fucoidan NPs potential carriers in oral delivery systems.

Park et al. prepared core/shell microparticles by co-axial electro-spray drying [167]. FF was mixed with the antioxidant α-lipoic acid (ALA). The size of the microparticles ranged from 5.4 to 8.4 µm. FF and ALA were detected within the core, and the chitosan within the shell of the microparticles. These composite microparticles were able to gel by water uptake and then swelled, contrary to the physical mixture of FF and chitosan; the swelling was found to depend on pH with a decrease for pH values higher than 7. In the same way, decreasing the chitosan/FF ratio lowered the swelling of the hydrogel. Finally, the release behavior of ALA from the gel was validated in vitro in different pH media by applying different electric potentials, inducing the drug release. The cumulative amounts of released ALA were quantified over 48 h to conclude that not only a declining concentration gradient occurred but also that the physical gelation between FF and chitosan over time reduced the diffusion of ALA, resulting in a unique release behavior with possible applications in drug delivery systems, wound healing dressings or scaffolds.

Lee et al. combined the immunotherapeutic activity of an acetylated FF with self-organized nanospheres loaded with doxorubicin (DOX) [171]. FNPs reached a 71% loading efficiency and the release followed a first order kinetic. FNPs were incubated for 24 h with RAW-264.7 macrophages, then tumor necrosis factor α (TNF-α) and granulocyte-macrophage colony-stimulating factor (GM-CSF) expression levels were measured. TNF-α expression was improved by a factor of 1.13 and GM-CSF by a factor of 1.86 as compared to unloaded FNPs and free DOX in a multidrug resistant cell model. Finally, these FNPs were considered as good candidates for combined immunotherapy and chemotherapy.

In the development of an oral drug delivery system, chitosan was found to modulate the opening of the tight junctions of epithelial cells [177]. Da Silva et al. prepared fucospheres with anti-coagulant properties for oral delivery by a nanocoacervation [178]. The size of FNPs ranged from 198 to 352 nm mean diameter and their zeta potential was measured between 35 and 53 mV. The anticoagulant activity of aqueous suspensions of these fucospheres was not found significantly different from that of FF, and FNPs did not show cytotoxicity for Caco-2 cells up to 1 mg/mL after 3 h of incubation.

At the same time, Yu et al. designed fucospheres to release an over sulfated FF via the oral route [96]. FNPs were able to go through a Caco-2 cell monolayer by opening the tight junctions. Eventually, it was found that released over sulfated FF had a higher anti-angiogenic activity than native FF.

By mixing FF and soybean lecithin in a homogenizer, Kimura et al. prepared unilamellar liposomes mixed with FF (FFL) of 100 nm and compared their cytotoxic effects with the native FF on osteosarcoma in vitro and in vivo [172]. FFL were found to reduce the viability of human osteosarcoma cell line 143B in vitro with a maximum inhibition for 2 mg/mL of liposome and 72 h of incubation. In addition, FFL were more potent than FF to induce apoptosis in cells. Mice were inoculated with murine osteosarcoma LM8 tumor cells and treated with FFL or native FF. FFL induced a reduction of the volume and the weight of the tumor compared to FF-treated mice.

Lira et al. compared in 2011 the cytotoxicity on macrophages and fibroblast murine cell lines of FNPs obtained by coating poly(isobutylcyanoacrylate) (PIBCA) with a blend of dextran and FF with two methods, a redox radical emulsion polymerization (RREP) and an anionic emulsion polymerization (AEP) [188]. FNPs prepared by the former were four times less toxic than those prepared by the latter. The authors also observed that FNPs obtained by RREP were not stable with a ratio FF/dextran of over 25, while FNPs obtained by AEP were stable in suspension with 100% FF as coating material.

### 4.2. Fucoidans in Diagnostic Nanosystems

Nanosystems for diagnosis must be blood compatible and non-toxic at concentrations sufficient for recording relevant images of the region of interest. To a large extent, sulfated polysaccharides could meet these criteria as vectors of imaging markers. Among these, fucoidans have been evidenced as good candidates to image atherothrombosis in vivo [156,191], and still awaited are studies evidencing their usefulness for cancer imaging.

In 2011, Rouzet et al. showed the direct complexation of ^99m^Tc by a commercial FF allowing SPECT imaging of thrombosis and heart ischemia thanks to the interaction of FF with P-selectin overexpressed by activated platelet and activated endothelium [158]. Biodistribution studies of ^99m^Tc-labelled FF in rat by SPECT imaging evidenced a urinary elimination and a moderate liver and spleen uptake which decreased with a fraction obtained from treatment with calcium ions of FF [160].

With the same FF, Suzuki et al. evidenced the capacity of superparamagnetic FNPs to detect in vivo the intraluminal thrombus of abdominal aortic aneurysm in a rat model with a 4.7 T MR Imager [191]. FNPs were obtained by linking FF to the carboxymethyldextran shell of Ultrasmall Superparamagnetic Iron oxide (USPIO). FNPs had a size of 50 nm and a zeta-potential of −14.3 mV. Surface Plasmon Resonance experiments evidenced an affinity of the FNPs for P-selectin in 1–10 nM range compared to NPs coated only with carboxymethyldextran, in accordance with previous work of Bachelet et al. [55]. Other in vitro studies showed the capacity of these FNPs to bind to activated human platelets [156].

Bonnard et al. developed polysaccharide-based NPs from dextran and pullulan cross-linked with sodium trimetaphosphate (STMP) in a water-in-oil emulsion [157,161]. FF was added to the emulsion to provide NPs functionalized with fucoidans (FNPs) with an average hydrodynamic diameter of 358 nm and a zeta-potential of −16 mV. MPFs contained about 1.6% (*w/w*) of FF and energy dispersive X-ray (EDX) spectrum showed the presence of FF at the surface of the particles. The interaction of MPFs with activated human platelets was validated in vitro. MPFs were radiolabeled with ^99m^Tc [158] and used to image an aneurysmal thrombus in a rat model. Iron oxide embedded MPFs showed a high affinity for activated Human platelets in vitro and MR images of aneurysmal thrombus and activated endothelium were also obtained in murine models [159]. In another study, the authors developed MPFs containing USPIO for magnetic resonance imaging [159]. On animal models a significant contrast enhancement of thrombus was obtained from 30 min to 2 h after the injection of MPFs.

In 2014, Li et al. developed a contrast agent for PET imaging [162]. FF was labelled with gallium 68 to image vulnerable active atherosclerosis plaques expressing P-selectin. After the validation with in vitro and ex vivo studies, they localized atherosclerotic plaques on an apolipoprotein E–deficient mice model using PET imaging. Anatomic structures of plaque were confirmed by 17.6 T MRI to correlate their results. The P-selectin affinity PET tracer was found to discriminate active and inactive atherosclerotic plaques.

### 4.3. Fucoidans in Regenerative Medicine

Marine polysaccharides have been used for years to design scaffolds for tissue engineering due to their interesting bioactivities and their biocompatibility. Senni et al. reviewed the studies in this field [192]. Particularly, fucoidans have raised interest in the design of biocomposites, especially for bone tissue engineering. So it is not surprising to find now the most advanced developments in this domain although there are still comparatively very few studies.

In 2008, Changotade et al. treated a commercial bone substitute (Lubboc^®^) with a low molecular weight FF (LMWF) to improve bone regeneration [185]. The authors found out that the pretreatment of the bone substitute with LMWF promotes human osteoblast proliferation, collagen type I expression and favors alkaline phosphatase activity enhancing the mineralization of the bone tissue. Regarding the origin and structure of LMWF used, the authors refer to older works without specifying any product parameter used in their study.

Three years later, Jin et al. developed polycaprolactone (PCL)/fucoidan composite scaffolds for bone tissue regeneration [186]. PCL/FF scaffolds with a 300 µm pore size dramatically increased the hydrophilic properties (with ≥5 wt % of fucoidans). In addition mechanical properties were improved even with a low fucoidan/PCL ratio (as an example: a 22% increase of Young’s modulus at 10 wt % of fucoidans). The biocompatibility of the scaffolds was assessed on osteoblast-like-cells (MG63) evidencing a better cell adhesion to the surface of the FF-containing scaffolds with three times more mineralization compared to the pure PCL scaffold after 14 days of cell culture. At the same time, Lee et al. prepared a biocomposite of polycaprolactone (PCL) and FF [187]. The biocomposite showed a better distribution of osteoblast-like cells (MG63) compared to pure PCL mats. Furthermore, total protein content, alkaline phosphatase activity, and calcium mineralization were better and were higher with PCL/FF micro/nanofibrous mats suggesting that FF-complemented biocomposites would make good candidates for tissue engineering applications.

Since 2013, S. K. Kim’s group has been developing scaffolds from hydroxyapatite/polysaccharide-based nanocrystals for bone tissue regeneration [181,182,183]. Chitosan/alginate scaffold (CAS) and chitosan/alginate/fucoidan scaffold (CAFFS) were first prepared. CAFFS with a pore size of 56–437 nm improved cytocompatibility, proliferation, and alkaline phosphatase secretion of MG63 osteosarcoma cells as compared to CAS. In addition, protein adsorption and mineralization were two times greater with CAFFS, which was attributed to the negative charges of FF sulfate groups. Then, they prepared scaffolds from hydroxyapatite (HapS) and hydroxyapatite mixed with FF (HapFFS) to induce FGF-2 activity and angiogenesis [182]. HapFFS showed a mineralization effect two times higher than HapS. Scaffolds obtained more recently by mixing HapFFS with chitosan evidenced a better mineralization as well as a good biocompatibility with mesenchymal stem cells (PMSCs) likely due to a suitable micro architecture for cell growth and nutrient supplementation [183]. Note that no data about the FF were provided for the two first studies.

In 2015, Puvaneswary et al. prepared tricalcium phosphate-chitosan-fucoidan biocomposite scaffold and demonstrated the benefic effect of FF [184]. They showed that the addition of FF in the scaffold increased the release of osteocalcin allowing the osteogenic differentiation of human mesenchymal stromal cells in vitro. Furthermore, FF was found to improve the compression strength and the biomineralization of the scaffolds.

## 5. Discussion

Fucoidan-containing nanosystems were first developed for the delivery of different therapeutic agents [147] followed by studies on regenerative medicine and more recently on diagnostics. Most of them focused on structures obtained from a mixture of FF and chitosan, a cationic polysaccharide with a random alternation of β(1→4)-d-glucosamine, and *N*-acetyl-d-glucosamine. The formation of these nanosystems occurs from electrostatic interactions between sulfate and ammonium groups to generate multilayer architectures stable over a wide range of pH values and suitable for oral or parenteral administration. Different methods were used to obtain fucoidan-containing nanosystems such as emulsion, self-assembly, coacervation, polyelectrolyte complexing or ionic cross-linking, all without risks of modification of the polymer structure. Although, in some cases, fucoidans were used for their intrinsic biological properties, for most of these studies they appear to have been used more for an ability to form stable structures with chitosan, as well as for pre-supposed harmlessness. Interestingly, physicochemical data for chitosan are often more detailed than for FF for which they are in general limited and sometimes even absent. Indeed, as evidenced in Table 3, in most cases the origin of FF is the only information provided, and, as a consequence, it is difficult to compare the results. Only three studies provide sufficient characteristics to the readers, and additional works are needed for discussion [158,176,191]. On one hand, this lack of structural data does not allow drugs to be created based on these polysaccharides [193]. On the other hand, the developments for Human health improvements require well-defined reproducible fucoidan fractions. If not, the conclusions are unique for a particular fraction, and, as a consequence, the results cannot be reproduced.

Fucoidans are polysaccharides, one of the three families of natural macromolecules with proteins and nucleic acids. Scientists have been able to fully synthesize the latter two for several decades. However the complexity of fucoidan structures has significantly delayed this essential step in their development to Human health, and overall progress in this domain suffers from a lack of tools such as those that are readily available for studying nucleic acids and proteins. More generally, once a particular carbohydrate structure has been identified as being responsible for a biological effect, it often has to be synthesized in order to establish or confirm its structure assignment. Nevertheless, dedicated synthesis methods are time-consuming, limited to oligosaccharides, and practiced mostly by specialized laboratories using processes that may take months to years because of the structural complexity of these compounds. As a consequence, despite the prevalent role of polysaccharides and oligosaccharides in a wide range of biological processes, it is not surprising that there are so few carbohydrate based therapeutics and diagnostics on the market. In addition to monosaccharide-inspired drugs such as the influenza virus treatment Tamiflu (oseltamivir phosphate; Roche, Bâle, Switzerland), two drugs: acarbose (Precose, Glucobay; Bayer, Leverkusen, Germany) and heparin, stand out [194]. Note that both compounds were derived by isolation and reached the clinic before a detailed structure–activity relationship had been established. In particular, low molecular weight heparin (LMWH) (lovenox; Sanofi, Gentilly, France), mainly extracted from pig intestines and fractioned via chromatography, chemical cleavage or enzymatic hydrolysis, is still the only polysaccharide used in Human health since its first clinical trial reported in the early 80’s [195,196,197,198]. FF production follows the same process but the raw material is from vegetal origin, thereby preventing all contaminations attributed to animal products. However Health agencies have hardened the legislation about new pharmaceuticals in the last decade due to health scandals (in particular implicating LMWH in 2008 [199]), making FF more difficult to reach the market or even impossible without a reliable source. Anyway, scientists and companies who want to develop fucoidan-containing nanosystems up to clinical use must provide robust data about their product.

Nanomedicine approaches have revolutionized the treatment of human pathologies, in particular cancer and cardiovascular diseases [200,201]. Drugs are entrapped within sterically stabilized, long-circulating vehicles (therapeutics). Imaging markers such as radiolabels, USPIO or quantum dots allow real-time visualization of pathological areas (diagnostics). The theranostic strategy associates both types in unique structures. These tailor-made nanosystems are built from polymers, carbon nanosheets, lipids, metal oxides etc., sometimes mixed to get hybrid structures, shaped as spheres, rods, capsules or more complicated geometry, and surface-modified to improve their efficacy and decrease side-toxicity. Ultimately they can be grafted with ligands to target cellular/molecular components of the diseases [200,201]. Bioactive carbohydrates, and in particular fucoidan fractions, are good candidates thanks to their overall biocompatibility, high versatility with regard to chemical modifications, and relatively low production costs. However the clinical development of fucoidan-based biospecific systems for nanomedicine remains a challenge because it requires not only a translational approach involving a partnership with pharmaceutical companies and respecting specifications approved by Health agencies [202] but also implementing a secure process to obtain reliable fractions.

In this context, we have considered a rational approach in order to develop a clinical contrast agent using FF (see [55,156,158,191]). From the pioneer works of Varki et al. [75], P-selectin was confirmed as a relevant molecular target of a commercial FF (Ascophyscient^®^ from Algues & Mer, Ile d’Ouessant, France: a low molecular weight fucoidan fraction from *Ascophyllum nodosum*). In 2013, a joint laboratory was created with the Algues & Mer Company to secure the production of reproducible FF with well-defined composition and molecular weight. In 2015, these fucoidans were labeled by the French authorities as “raw materials for pharmaceutical uses”. Today, they are part of the European project Nanoathero for the development of a SPECT marker for human atherothombosis [203] and clinical trials will start soon.

## 6. Conclusions

Fucoidans are abundant polysaccharides with remarkable biological properties. Their vegetal origins (considering that fucoidans extracted from marine animals are a tiny part of the total amount), the absence of adverse effects, and an affordable price due to easy-to-handle production processes make them promising for Human health. However these advantages are also the main bottlenecks for developments in nanomedicine due to the difficulty in obtaining reproducible chemical structures and molecular weights from one batch to another. Up to now, fucoidans in nanomedicine have been mainly used for protein or drug delivery with few studies about medical imaging; applications to regenerative medicine being still limited to bone tissue regeneration in animals. So far, isolation from natural sources is the only effective way to get fucoidans, but it is no longer possible to consider the molecular weight together with l-fucose and sulfate contents of a bioactive fraction as the only relevant parameters for further developments. The use of fucoidans in nanomedicine will be legitimated only by a translational strategy from a reproducible starting material with a defined and reproducible structure. This goal can be achieved only via two ways: (i) validation of an industrial production from natural extracts; or (ii) total synthesis with enzymes or chemical reactions. Currently, the first way is available; the second one is likely within the next decades [45]. The biomedical market represents an enormous opportunity for fucoidans, as their potential added value can, in principle, justify the inherent risk related with the development and approval of such products. Moreover, the possibility of developing a wide variety of chemically modified derivatives makes fucoidans versatile materials that could be applied in other fields of technological interest. This is a continuing challenge to polymer and biomaterial scientists, but it is already possible to anticipate that these strategic approaches will widen up perspectives and potential applications in the future.

## Figures and Tables

**Table 1:** Biological properties of fucoidans and identified targets.

Biological Properties	Identified Targets	References
Anticoagulant/anti-thrombotic	Antithrombin, heparin cofactor II	[11,34,57,58,59]
Anti-complement	C4, C4b,2a, C3, and C3b,Bb	[56,59,60]
Anti-viral	CD4	[61,62,63,64,65,66,67,68]
Anti-inflammatory	P-selectin and L-selectin	[54,55,59,69,70,71,72,73,74,75,76]
Angiogenic effect	VEGFs, bFGF, FGF-2//α6, β1, and PECAM-1 integrin subunits	[10,11,54,59,77,78,79,80,81,82,83,84,85,86,87]
Anti-cancer	Capsases-3, -8 and -9, MAPK and their inhibitors, HIF-1	[29,88,89,90,91,92,93,94,95,96,97,98,99,100,101,102,103,104,105,106,107,108,109,110]
Anti-diabetic	α-glucosidase, α-amylase	[111,112,113,114,115,116,117,118]
Immune potentiating	NK cells, T-cells, dendritic cells	[119,120,121,122,123]
Antioxidant	-	[124,125,126,127,128,129,130,131,132,133,134,135,136,137,138,139,140,141]

**Table 2:** Applications of fucoidan-containing nanosystems in nanomedicine.

Application	References
Imaging agent	[156,157,158,159,160,161,162]
Protein delivery	[163,164,165,166,167]
Small drug delivery	[168,169,170,171,172,173,174,175,176]
Anti-coagulant	[177,178]
Gene delivery	[179,180]
Regenerative medicine	[181,182,183,184,185,186]

**Table 3:** Features of the fucoidan fractions used in nanomedicine related studies.

Study	Objective	Origin of Fucoidans	Molecular Weight	Sulfate Content *	Other Data	Remarks
Bonnard et al. [157,159]	P-selectin tageting FMPs for SPECT imaging	*F. vesiculosus*	57 kDa/23 kDa	-	-	Commercial fucoidans from Sigma Aldrich Company
Changotade et al. [185]	Pretreatment of bone tissue substitute	*-*	-	-	-	-
Da Silva et al. [178]	FNPs preparation for therapeutic purposes	*F. vesiculosus*	-	-	-	Commercial fucoidans from Sigma Aldrich Company
Huang et al. [169]	Gentamicin controlled release	*F. vesiculosus*	-	-	-	Commercial fucoidans from Sigma Aldrich Company
Huang et al. [174]	Curcumin controlled release	*F. vesiculosus*	-	-	-	Commercial fucoidans from Sigma Aldrich Company
Huang et al. [165]	FGF-2 controlled release with FNPs	*F. vesiculosus*	80 kDa	-	-	Commercial fucoidans from Sigma Aldrich Company
Huang et al. [166]	SDF-1 controlled release with FNPs	*F. vesiculosus*	80 kDa	-	-	Commercial fucoidans from Sigma Aldrich Company
Jeong et al. [182]	Design of a scaffold for bone tissue regeneration	-	-	-	-	-
Jin et al. [186]	Design of a scaffold for bone tissue regeneration	*U. pinnatifida*	-	-	-	Commercial fucoidans from Haewon Biotech Company
Kimura et al. [172]	Evaluation of cytotoxic effects of FNPs	*C. okamuranus*	2–10 kDa	-	-	Fucoidans extracted and purified by the authors
Kurosaki et al. [180]	DNA delivery with FMPs	-	-	-	-	Commercial fucoidans from Sigma Aldrich Company
Lee et al. [171]	DOX controlled release with FNPs	*F. vesiculosus*	-	-	-	Commercial fucoidans from Sigma Aldrich Company
Lee et al. [187]	Electrospun mats for Tissue engineering	*U. pinnatifida*	-	34.2%	62.12% total polysaccharide	Commercial fucoidans from Haewon Biotech Company
Li et al. [162]	P-selectin tageting FMPs for PET imaging	*-*	-	-	-	Commercial fucoidans from Sigma Aldrich Company
Lira et al. [188]	Preparation and evaluation of FNPs	*S. cymosum*	53 kDa	-	-	Fucoidans extracted and purified by the authors
Lowe et al. [183]	Design of a scaffold for bone tissue regeneration	*F. vesiculosus*	-	-	-	Commercial fucoidans from Sigma Aldrich Company
Nakamura et al. [164]	FGF-2 controlled release	*K. crassifolia*	-	-	-	Fucoidans extracted and purified by the authors
Park et al. [167]	ALA controlled release with FMNs	-	-	-	-	Commercial fucoidans from Haewon Biotech Company
Pinheiro et al. [176]	PLL controlled release	*F. vesiculosus*	57.26 kDa	-	40.2% Fuc, 2.98% Xyl, 0.55% Man, 3.6% Gal, 9.17% Ur.Ac, 0.11% Rha, 0.21% Glu	Commercial fucoidans from Sigma Aldrich Company
Puvaneswary et al. [184]	Design of a scaffold for bone tissue regeneration	*F. vesiculosus*	-	-	-	Commercial fucoidans from Sigma Aldrich Company
Sezer et al. [179]	DNA delivery with FMPs	*F. vesiculosus*	80 kDa	-	-	Commercial fucoidans from Sigma Aldrich Company
Sezer et al. [189,190]	FNPs for dermal burns treatment	*F. vesiculosus*	80 kDa	-	-	Commercial fucoidans from Sigma Aldrich Company
Suzuki et al. [191]	P-selectin targeting FNPs for MRI imaging	*A. nodosum*	8 kDa	27%	45% l-fucose, 25% d-glucuronic acid	Commercial fucoidans from Algues et Mer Company
Venkatesan et al. [181]	Design of a scaffold for bone tissue regeneration	-	-	-	-	-
Wu et al. [175]	Berberine controlled release	*-*	80 kDa	-	-	Commercial fucoidans from NOVA Pharma & Liposome Biotech Company
Yu et al. [168]	Berberine controlled release	*L. japonica*	-	24.3%	3.5% carboxyl groups	Commercial fucoidans from NOVA Pharma & Liposome Biotech Company
Yu et al. [96]	Oversulfated FF release via oral route	*F. vesiculosus*	80 kDa	41.7%	-	Commercial fucoidans from NOVA Pharma & Liposome Biotech Company

* g/100 g.
